# Pheromone relay networks in the honeybee: messenger workers distribute the queen’s fertility signal throughout the hive

**DOI:** 10.1186/s12915-024-02083-w

**Published:** 2024-12-18

**Authors:** Thomas O. Richardson, Tomas Kay, Laurent Keller, Nathalie Stroeymeyt

**Affiliations:** 1 School of Biological Sciences, University of Bristol, Bristol, UK; 2 Department of Ecology and Evolution, University of Lausanne, Lausanne, Switzerland; 3 Social Evolution Unit, Cornuit 8, BP 855, 1885 Chesières, Ollon, Switzerland

**Keywords:** Social insect, Animal communication, Fertility signalling, Queen pheromone, Transmission, Contagion, Contact network, Automatic tracking

## Abstract

**Background:**

The harmonious operation of many insect societies depends upon colony-wide dissemination of a non-volatile pheromone produced by a single queen, which informs workers of her presence. This represents a major challenge in large colonies. Honeybee colonies, which can exceed 60,000 bees, are believed to solve this challenge using ‘messenger’ workers that actively relay the queen pheromone throughout the hive. However, little is known about the structure and effectiveness of the underlying relay network or the biology of messaging.

**Results:**

Here, we combine automated tracking with modelling to address these outstanding questions. We find that both queen movement and worker messaging play fundamental roles in queen pheromone dissemination. Fine-grained analyses of worker behaviour confirmed the existence of active messaging, as physical contacts with the queen caused workers to move faster and straighter, thereby accelerating pheromone transmission. Finally, we show that messaging follows a stereotypical developmental trajectory, resulting in an age-dependent hierarchical relay network, with the most intense messaging observed between three and five days of age, when workers undergo a suite of physiological changes associated with queen rearing.

**Conclusions:**

These results suggest that the individuals that contribute most to advertising the presence of the queen are also the ones that control queen production.

**Supplementary Information:**

The online version contains supplementary material available at 10.1186/s12915-024-02083-w.

## Background

The societies of ants, bees and wasps are typically characterised by a cooperative division of reproductive labour in which queens lay eggs, and workers raise the brood and perform other essential tasks such as foraging. In the presence of a fecund queen, the fitness of both the queen and the workers is generally increased when workers rear the queen’s offspring (i.e., their sisters) rather than laying their own eggs [1]. However, the loss of the queen dramatically alters the optimal behavioural strategy of the workers. Following queen death, worker fitness is maximised by laying eggs themselves, and/or by increasing the proportion of larvae that develop into queens rather than workers [2]. Worker fitness is therefore dependent on their ability to rapidly detect the death or waning fecundity of the queen.

In species that form small colonies, the queen can communicate her presence to workers by direct contacts with all workers and possibly also through dominance interactions [3, 4]. However, in species that form large colonies, such as the honeybee, the direct-encounter strategy is not feasible [5, 6]. Rather than attempting to contact each worker directly, a mated honeybee queen advertises her presence using a chemical fertility signal: a non-volatile blend of at least nine hydrocarbons [7–11], which is produced in the mandibular glands, and which coats the surfaces of the head, thorax and abdomen. This chemical blend (henceforth, ‘queen pheromone’) is gathered by young workers, which assemble in a ring-shaped ‘court’ or ‘retinue’ around the queen, excitedly antennating and licking her body [5, 12]. Small amounts of queen pheromone are transferred onto these workers and then passed on to nestmates as these workers move through the hive [13–16]. Under normal circumstances, this relay network is so effective that changes in worker behaviour become apparent within thirty minutes of the loss of the queen [5, 17]. However, it has also been suggested that communication over the relay network breaks down when the worker population grows so large and congested that the pheromone ceases to reach the entire workforce, and that this triggers colony reproduction by swarming [12, 18]. As swarming can lead to the loss of up to half of the workforce, and thereby severely reduce colony productivity, the relay network structure has direct consequences for both commercial apiculture and general honeybee ecology. However, as previous studies of honeybee queen fertility signalling have relied on painstaking manual observation of a few focal individuals, or of entire cohorts of same-age bees [5, 13, 14, 16, 19–25], very little is known about the structure and transmission properties of the relay network.

A previous study of queen fertility signalling in a standard two-frame observation hive suggested that bees that recently contacted the queen by participating in the retinue (hereafter ‘post-retinue’ bees), act as active ‘messengers’ that hasten the transmission of the queen pheromone to their nestmates by performing excited wide-ranging runs throughout the hive [14]. However, because these results were based on comparisons between post-retinue and randomly-selected and (approximately) age-matched control bees, rather than individual-level comparisons of behaviour before versus after queen contact, the possibility remains that the reported greater excitation of the post-retinue compared to the controls was due to sampling effects, rather than the causal effect of the queen encounter [23]. Thus, conclusive evidence that workers actively alter their behavior following queen encounters in a way that enhances the spread of the queen pheromone is still lacking.

To overcome these difficulties, we glued unique barcode-like tags to the thoraces of 10,893 zero-day old ‘callow’ workers of the honeybee *Apis mellifera*, and introduced them into ten queenright colonies housed in observation hives consisting of a single, double-sided frame. As social insect workers exhibit predictable age-associated changes in behaviour [26–29], the callow introductions were staggered into eight successive cohorts spanning 21 days, so that by the last cohort, the tagged workers constituted a demographically mixed population. We used automated tracking [30–32] to record the tagged bees’ spatial trajectories for seven days (Additional File 1: Table S1). As the queen pheromone is transferred mainly when bees lick or antennate one another, we used a geometric approach to identify the head-to-head and head-to-body contacts between bees [30]. To investigate how these encounters support pheromone propagation, we used previously-published transmission rate estimates to model transmission over the time-ordered sequence of bee-to-bee encounters. These relay networks were then analysed to (i) explore how queen movement influences worker exposure to the queen pheromone, (ii) quantify the role of indirect (worker-to-worker) transmission, (iii) test whether queen encounters cause workers to exhibit behavioural changes consistent with active ‘messaging’, (iv) assess whether these changes speed up transmission, and (v) identify the developmental stage when workers exhibit peak messaging.

## Results

### Queen behaviour influences pheromone transmission

**Fig. 1:**
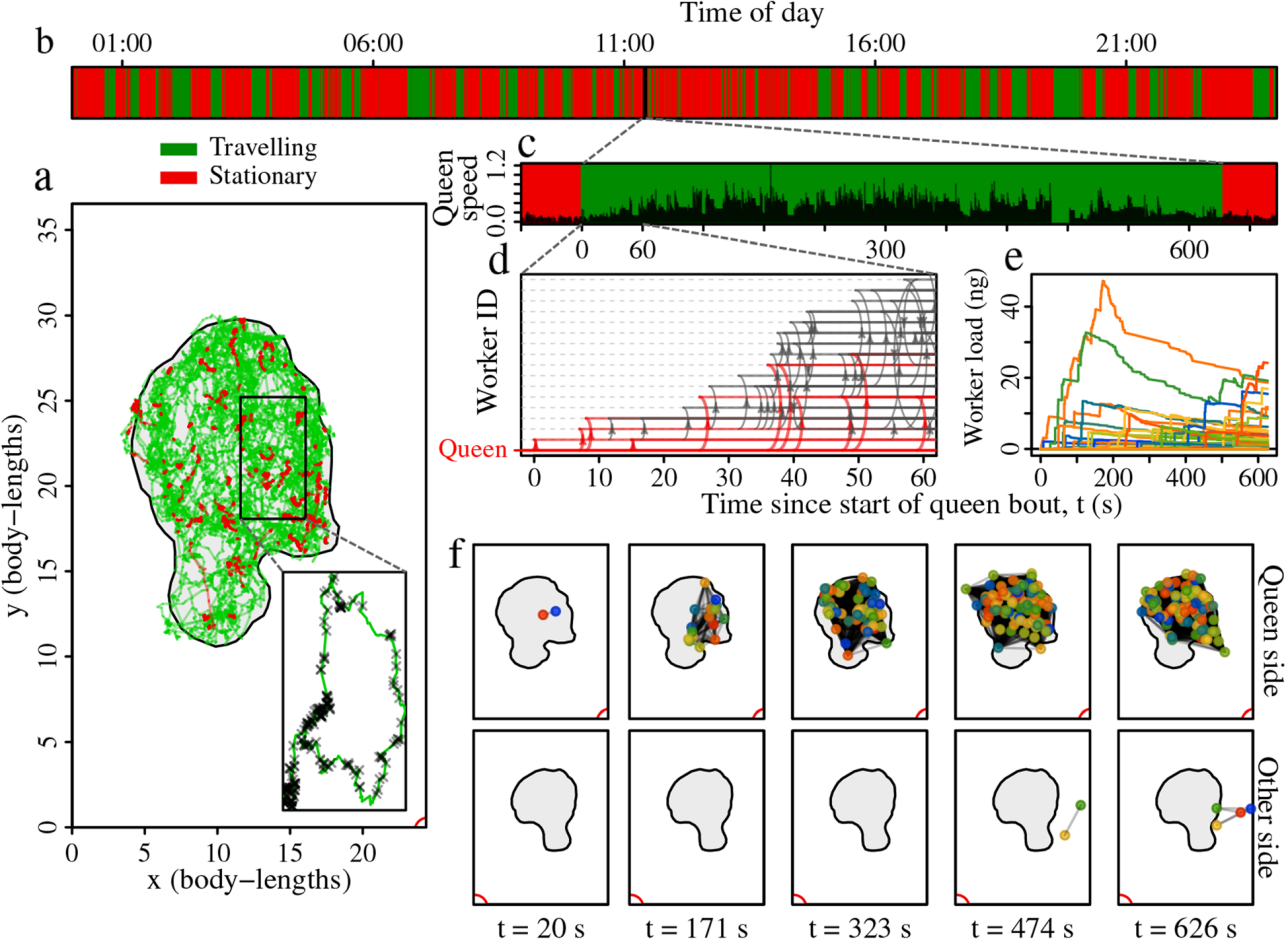
Quantifying the influence of queen behavioural state on pheromone transmission. **a** An example 24-hour queen trajectory on one side of the hive; travelling segments are shown in green and stationary segments in red. The black polygon indicates the boundary of the broodnest and the corner marked with a red arc represents the nest entrance. The blow-up shows the queen’s movement during an example 10-minute travelling bout (focal bout). Crosses indicate encounters with workers. **b** Behavioural state sequence of the queen during the same 24 hours, inferred by applying a HMM to the queen’s trajectory. The black line highlights the focal bout. **c** Queen movement speed during the focal bout (units are square-root-transformed body-lengths/sec). **d** Transmission sequence during the first minute of the focal bout. Each line on the y-axis represents a different bee, with the queen at y=0, and with the workers positioned according to the transmission order. Red and grey arrows respectively depict queen-to-worker and worker-to-worker pheromone transfers. **e** Individual-level load dynamics for the entire bout. **f** Snapshots of the spatial coverage of the informed workers on both sides of the hive. Points show locations of informed workers, lines show pairwise distances

As the mobility of the honeybee queen has been suggested to play a critical role in the transmission of the queen pheromone and the rearing of new queens [5, 18, 33], we first investigated how the queen’s mobility patterns influenced how broadly the queen pheromone spreads through the colony. Previous studies have found that the movement of workers in several species of social insects [29], and also of honeybee queens [14] can be classified into two states: one characterised by local, area-restricted movement, and another characterised by wide-ranging movement. To identify these states, we used the *moveHMM* package [34] for *R* to fit a two-state hidden Markov model (HMM) to each of the 7 daily trajectories of the 10 queens. Across all 70 daily trajectories, the HMMs consistently revealed that queens frequently switch between two states, one associated with slow movement and large turn angles, and another associated with more rapid movement and smaller turn angles, which we labelled ‘stationary’ (S) and ‘travelling’ (T), respectively (Fig. 1a-b, Additional File 1: Fig. S1, Table S2).

We next investigated how the queen’s behavioural state influenced her physical encounters with workers. As the encounter rate of an agent within a population generally depends upon its speed [35], we expected that queens would experience a higher encounter rate when travelling than when stationary. Consistent with this expectation, and with previous results [19, 24], the encounter rate of queens was higher in the travelling than in the stationary state (Additional File 1: Fig. S2; Linear Mixed-Effect Model (LMM) with day & replicate as random effects, effect of state on encounter rate: d.f.=1, $\chi ^2$=123, *p*<0.0001). Furthermore, encounters were significantly shorter in the travelling than in the stationary state (Additional File 1: Fig. S2; LMM, effect of state on encounter duration, d.f.=1, $\chi ^2$=116, *p*<0.0001).

To explore how these state-dependent encounter patterns influence queen pheromone transmission, we developed an individual-based model in which queen pheromone spreads via both direct (queen-to-worker) and indirect (worker-to-worker) physical encounters (see Methods). The model was inspired by models of information flow [36] and disease transmission [37] over time-ordered contact sequences, and was parameterised using published empirical data on queen pheromone transmission [5, 16, 38]. In this model, we assumed that (i) there is a single inexhaustible and constant source of pheromone: the queen [16], (ii) pheromone transmission occurs during contacts in which a receiver bee antennates and/or licks the body of a pheromone-carrying bee (queen or pheromone-carrying worker), (iii) the amount transmitted depends on the contact’s duration and on the proportion of antennation versus licking carried out by the receiver, and (iv) the pheromone attenuates through decay [39] and ingestion [16]. A full description of the model and its parameterisation can be found in Supplementary Information (Additional File 1).

**Fig. 2:**
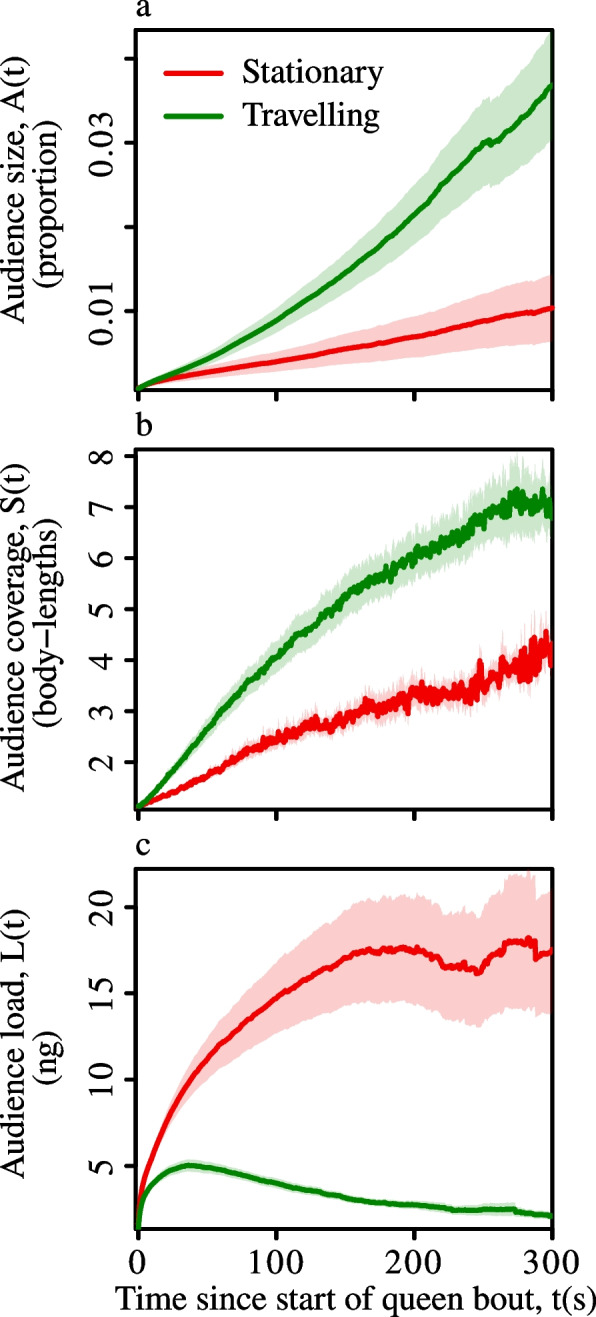
Short-term dynamics of queen pheromone transmission. Panels show the growth in **a** audience size (proportion of the colony that are informed), **b** average spatial separation between informed workers, and **c** average pheromone load of informed workers, for stationary versus travelling queen bouts. Lines & shaded areas show the cross-colony grand mean & standard error. Each colony contributes a single value to the grand mean (n=10)

We then ran the transmission model on all bee-to-bee contacts that occurred between the start and end of each queen movement bout (Fig. 1c-e), recording the growth in the ‘audience’ of informed bees over time. A worker was defined as an audience-member if its current pheromone load exceeded a sensitivity threshold [38], regardless of whether the pheromone was acquired from the queen or from other workers (see Methods section). The audience size grew faster for travelling bouts than for stationary bouts (Fig. 2a). Furthermore, measuring the pairwise Euclidian distances between all bees in the audience (Fig. 1f) revealed that area covered by the audience increased more quickly for travelling than for stationary bouts (Fig. 2b). On the other hand, during stationary bouts the audience typically received a higher pheromone load than during travelling bouts (Fig. 2c). Considering that queens allocate roughly similar times to each state (time stationary, grand mean ± standard error = 58±2.4%, calculated across the 10 colony means), these results raise the possibility that queen’s frequent switches between bouts of stationary and travelling behaviour may help balance the size of their audience against the quantity of pheromone transferred to each audience member.

### Colony-wide pheromone spread via direct and indirect transmission

**Fig. 3:**
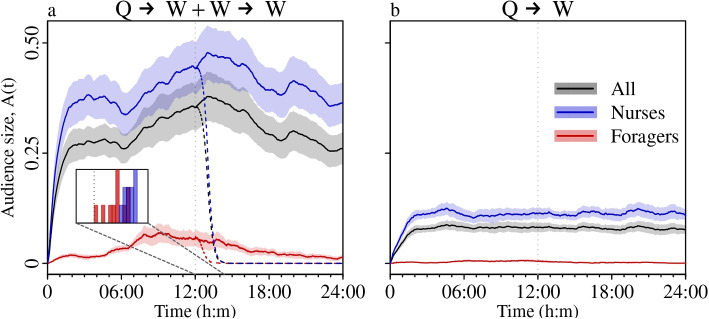
Indirect transmission enhances queen pheromone spreading, and nurses are more exposed than foragers. Growth curves show the proportion of informed bees as a function of time for daily contact sequences including both direct and indirect transmission (**a**, $Q \rightarrow W + W \rightarrow W$), or in the absence of indirect transmission (**b**, direct transmission only, $Q \rightarrow W$). Lines and shaded areas show the cross-colony grand means and standard errors, respectively. Each colony contributes a single value to the mean (n=10). Dashed coloured lines indicate the decay in the proportion of informed workers after the simulated removal of the queen at 12:00. The histograms in panel (**a**) show the post queen removal half-life distributions for informed nurses (blue) and foragers (red)

To evaluate how the queen pheromone spreads through the colony over longer timescales, we ran the transmission model on the full daily contact sequences. As young nurse workers are thought to be responsible for provisioning the queen, detecting the loss of a queen and raising new queens [14, 20, 40–42], we aimed to test whether different worker groups are more or less exposed to the queen pheromone. To do so, we applied a ‘soft’ community detection algorithm (FacetNet [43]) to the daily bee-to-bee contact networks [28]. This revealed that the contact networks contained three partially overlapping communities (Additional File 1: Fig. S3), namely, two nurse groups ($N_A$ & $N_B$, corresponding to the two patches of broodnest cells on either side of the hive [29]), and the foragers (*F*). As FacetNet uses scores in the range 0–1 to quantify the affiliations of each individual to each community, we defined nurses as bees that scored $(N_A + N_B) {>}^{\ 2\!}/_{3}$, and foragers as those with $F {>}^{\ 2\!}/_{3}$. We then tracked the proportions of each group in the ‘informed’ state over the course of the day. Averaging across these daily growth curves revealed that after an intial phase of rapid growth, the proportion of nurses in the informed state plateaued at around 40%, whereas that of informed foragers never exceeded 10% (Fig. 3a, blue versus red lines). This indicates that queen pheromone is heterogeneously distributed across workers, with nurses – individuals that are the most involved in queen-related tasks – being more exposed than foragers.

Previous work on how bees respond to the loss of the queen has focused on either worker behaviour (e.g., changes in aggression, mobility, and acoustic signalling) or colony-level processes (e.g., construction of replacement queen cells), and consequently the existing reaction time estimates vary from under an hour [5, 17], to close to a day [44, 45]. To evaluate whether our model produces realistic estimates for the dwindling – and eventual disappearance – of the informed workers after queen loss, we performed an *in-silico* queen removal experiment. Daily time-stamped contact sequences were edited to remove all contacts between the queen and workers that occurred after midday, and the transmission model was then run on these edited sequences. This showed that queen removal precipitates a rapid decay in the population of informed workers (Fig. 3a, dashed lines). Furthermore, there were differences in the half-lives of the population of informed bees for the two task groups: the population of informed foragers took 36±5 minutes to shrink to half its initial size (mean±standard error, n=10), whereas the informed nurses took 61±3 minutes (paired t-test, nurse versus forager half-life, d.f.=9, t=4.9, p=0.0009; Fig. 3a, histograms). As well as reinforcing the notion that changes in worker behaviour following queen loss are a direct response to the sudden reduction in queen pheromone flow over the contact network, the close agreement between these half-life times and previous reports of workers reacting in under an hour [5, 17] suggests that our simulation model generates reasonable approximations of real-world queen pheromone transmission.

Finally, to assess the importance of indirect transmission (or messaging) by workers in advertising the queen’s continuing presence, we created daily contact sequences in which only direct transmission was possible (Q$\rightarrow$W contacts only) by removing all of the worker-to-worker contacts. We then ran the transmission model on these reduced contact sequences, and compared the audience growth curves with those from the original, full contact sequences (Q$\rightarrow$W + W$\rightarrow$W contacts). These comparisons revealed that the absence of indirect transmission led to a reduction in the size of the queen-informed audience from $\sim$ 25–40% (Fig. 3a, black line), to less than 10% of the colony (Fig. 3b, black line). Furthermore, in the absence of indirect transmission (Q$\rightarrow$W contacts only), the proportion of informed nurses dropped to $\sim$10%, whilst the proportion of informed foragers was close to zero (Fig. 3b, blue and red lines). This difference indicates that indirect transmission via workers plays a fundamental role in disseminating the queen pheromone throughout the colony, and thus that pheromone-carrying workers play a role akin to relay nodes in communication networks.

### Workers orient towards high-load messengers

**Fig. 4:**
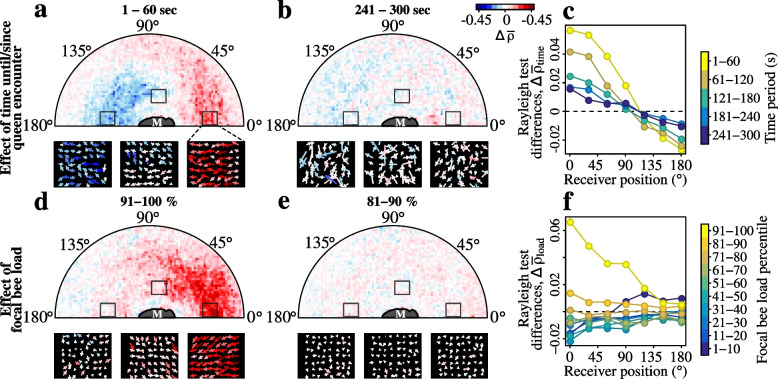
Workers orient towards nestmates that recently encountered the queen, or that have high queen pheromone loads. **a**-**b** Difference maps showing the change in the orientation strength of workers around a messenger bee (M). Worker orientation strengths are quantified using the Rayleigh test statistic, $\rho$. Panels **a** & **b** show respectively the post-pre differences in receiver orientation strengths for the first and fifth minutes either side of the queen contact (i.e., $\Delta \bar{\rho }_{time} = \bar{\rho }_{1:60} - \bar{\rho }_{-1:-60}$ & $\bar{\rho }_{241:300} - \bar{\rho }_{-241:-300}$). Arrow plots show post-pre difference vectors, $\Delta \vec v$, for areas to the front, the side, and behind the messenger. **c** Angular transect of the post-pre orientation strength differences as a function of the position of the receiver worker. Positions are measured clockwise relative to the heading of the messenger. Dots and error bars represent means & standard errors. **d**-**e** Difference maps comparing worker orientation strength toward a post-retinue messenger (M) with a given queen pheromone load, versus all post-retinue bees irrespective of their pheromone load (i.e., $\Delta \bar{\rho }_{load}$). Panels **d** & **e** show respectively the difference maps for messengers with a load in the top, and the second deciles (i.e., $\rho _{91:100} - \rho _{1:100}$ & $\rho _{81:90} - \rho _{81:90}$). **f** Angular transect of the differences in receiver orientation strengths as a function of the position of the receiver

As our simulation results point to a potentially important role of messenger workers in spreading the queen pheromone, we next investigated the responses of receiver bees to pheromone-carrying workers. As the formation of the queen retinue is based on the ability of workers to sense and orient toward the queen [5, 22, 39], we expected to detect similar orientation responses towards workers carrying high pheromone loads. However, as we could not directly measure the actual load carried by each worker, we used two load proxies. The first was the time before and after each encounter with the queen; if queen pheromone is transferred during such encounters, then workers should orient more strongly toward nestmates that recently encountered the queen than toward the same bee in the moments just before it encountered the queen. As the following analyses concern how receivers orient towards individuals that are about to encounter the queen (i.e., enter the retinue), or that have recently encountered her (i.e., left the retinue), we refer to such individuals as ‘pre-retinue’ and ‘post-retinue’ bees respectively. We used the Rayleigh test statistic $\rho$ [46] to quantify the orientations of receiver bees within two body-lengths of a pre- or post-retinue nestmate, and then to visualise how the queen encounter caused these orientations to change we constructed ‘difference maps’ comparing, for example the orientations in the first minute after the queen encounter with the last minute before the encounter (Fig. 4a-c).

The difference map comparing the first minute after the queen encounter, with the last minute before it (Fig. 4a) contained a crescent-shaped zone around the head of the focal bee. Bees in this zone displayed a greater tendency to face toward a post-retinue bee than a pre-retinue bee (i.e., $\Delta \rho _{time}>$ 0). The minute-one difference map also contained a smaller zone around the abdomen in which nestmates tended to orient less toward a post-retinue bee than toward a pre-retinue bee (i.e., $\Delta \rho _{time} <$ 0, blue arrows, Fig. 4a). These effects declined as the time since the queen encounter increased (Fig. 4b-c).

Our second load proxy was the dynamical pheromone load estimates for individual workers, produced by running the transmission model on the daily contact sequences. If bees react to pheromone-bearing nestmates in the same way that they react to the queen herself [39], then we expected that workers should orient more strongly towards post-retinue bees with high simulated pheromone loads, than towards post-retinue bees with low simulated loads. To test this, we used the dynamical estimates of the queen pheromone loads of each bee to assign post-retinue bees to one of ten groups (i.e., the 1^*st*^, 2^*nd*^, ..., 10^*th*^ load deciles), and then calculated the difference between workers’ orientation strengths toward bees belonging to a given load decile, versus toward all bees. These difference maps showed that bees tended to face toward the head of a post-retinue nestmate with the very highest loads, that is, only when the load was in the top 10% of all bees in the colony (Fig. 4d-f). As with the previous time-based load proxy, the attraction was greatest for workers positioned around the head of the post-retinue bee. These behavioural assays are consistent with worker messaging, as workers preferentially reacted to nestmates that recently encountered the queen, and reacted more strongly the higher the pheromone load of the messenger.

### Queen encounters induce temporary worker excitation

We next tested whether post-retinue workers exhibit increased mobility and increased long-range movement, which would enhance the dispersal of the queen pheromone throughout the hive [14, 23]. To do so we used individual-level comparisons of the movement of bees just before (‘pre-retinue’) and just after (‘post-retinue’) they encountered the queen.

**Fig. 5:**
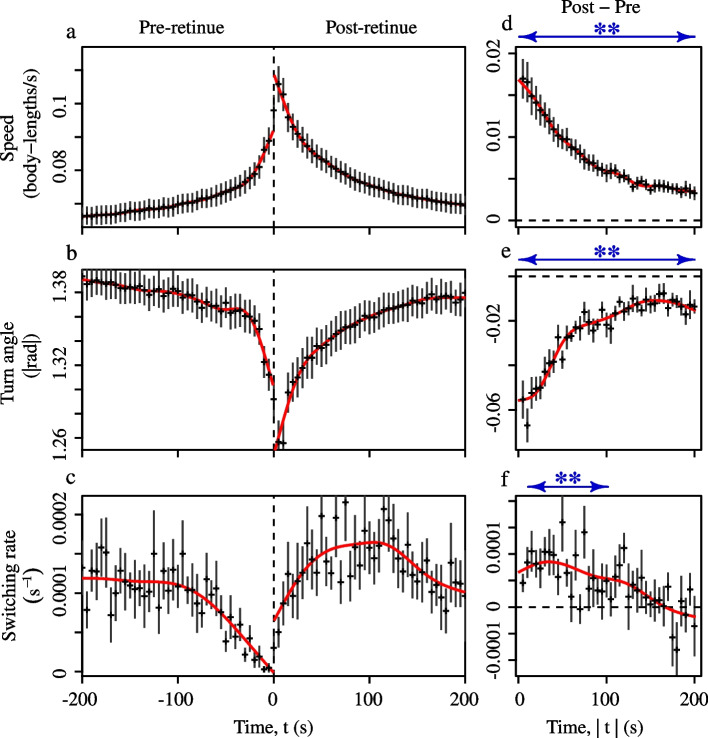
Physical contact with the queen induces worker excitation. **a**-**c** Worker movement before a physical encounter with the queen (‘Pre retinue’, negative times), and after it (‘Post retinue’, positive times). **d**-**f** Differences between the movement of pre- versus post-retinue workers. Differences were calculated by subtracting the observation at a given time *before* the queen contact, $T=-t$, from the observation at the same time *after* the contact, $T=+t$. Black crosses indicate cross-colony grand means & standard errors. Each colony contributes one value to the grand mean (*n*=10). Solid red lines indicate fits from the GAMMs described in the text. Blue arrows in **d**-**f** indicate times when the pre- and post-retinue GAMM fits were significantly different (at *p*<0.01)

Workers that had recently encountered the queen displayed increased mobility, moving significantly faster and straighter than they did before the encounter (Fig. 5a-b; Generalised Additive Mixed Models (GAMM) with day, replicate and worker ID as random effects, effect of ‘Post’ vs. ‘Pre’, instantaneous speed: $\beta$=0.007±0.00005, t=151, *p*<0.0001; unsigned turn angles, $\beta$=−0.024±0.0005, t=−47, *p*<0.0001). These differences persisted for $\sim$4 minutes after the end of the queen contact (Fig. 5d-f). Furthermore, compared to pre-retinue bees, post-retinue bees displayed a slightly higher probability of switching from one side of the frame to the other (Fig. 5c; GAMM, effect of ‘Post’ vs. ‘Pre’ on switching rate: $\beta$=0.000045±0.0000055 $\text {sec}^{-1}$, t=8.2, *p*<0.0001), although this effect disappeared after $\sim$2 minutes (Fig. 5f). Taken together these results show that queen encounters cause workers to exhibit both increased mobility, and an increased tendency to undertake wide-ranging excursions throughout the nest, consistent with the suggestion that pheromone-carrying workers act as messengers that actively modulate their behaviour to advertise the presence of the queen [14, 23].

### Post-retinue worker excitation boosts queen pheromone transmission

**Fig. 6:**
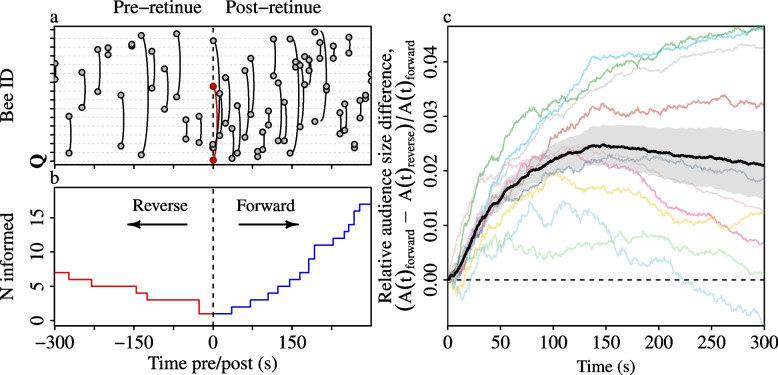
Comparison of forwards versus reverse-time transmission. **a** Example contact sequence covering a five minute pre- and post-retinue period. Individual bees occupy fixed positions on the y-axis, and physical encounters between pairs of bees are indicated by curved links. The queen is indicated in red. **b** Growth curves obtained from running the transmission simulation on the five-minute post-retinue contacts (‘forward’), and on the time-reversed five minute pre-retinue contacts (‘reverse’). **c** Relative differences between the audience size growth curves for the original post-retinue contact sequence versus the time-reversed pre-retinue contacts. Coloured lines indicate individual colony means. The black line and the shaded area indicates the grand mean & standard error, towards which each colony contribute a single value (*n*=10)

A fundamental assumption of the worker messaging hypothesis is that the behavioural changes exhibited by workers after encountering the queen should boost the spread of the queen pheromone [14]. To test this, we ran the transmission model on all bee-to-bee contacts that occurred within a five minute window following each encounter between the queen and a worker, recording the growth in the number of informed bees over this period (Fig. 6a). Then we compared those post-retinue growth curves with growth curves obtained by running the model over the time-reversed [47] pre-retinue contacts, that is, those that occurred during the five-minute window immediately preceding the queen encounter. In agreement with the messaging hypothesis, the growth curves derived from the post-retinue contacts outstripped those for the pre-retinue contacts, and this was the case in all 10 colonies (Fig. 6c). Hence, encounters with the queen induce behavioural changes in workers which enhance the onwards transmission of the queen pheromone, for example via increased contact rates with uninformed nestmates.

### Messaging follows a stereotyped developmental trajectory

**Fig. 7:**
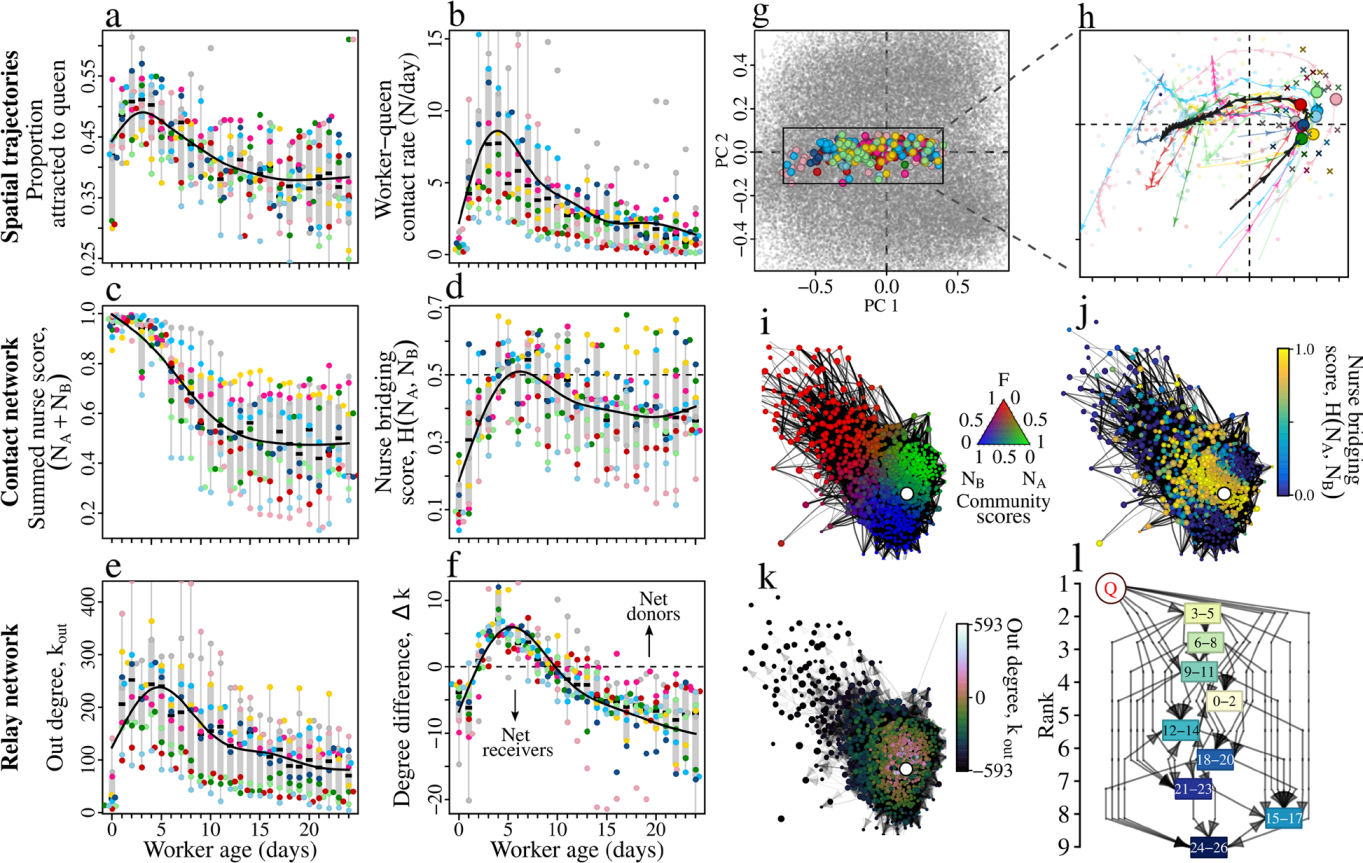
Expression of the messaging ‘syndrome’ follows a stereotypical developmental trajectory. **a**-**b** Active attraction of workers to the queen and contact rates between the queen and workers as a function of worker age. Points represent colony means, and point colours indicate colony identity. Solid lines indicate general additive models, fitted to the colony means. **c**-**d** Community affiliations in the daily contact networks as a function of age. **e**-**f** Spreading roles in the daily relay network according to age. Out-degree $k_{out}$ represents the number of nestmates an individual donates queen pheromone to, and degree difference $\Delta k$ represents the out-degree minus the in-degree (number of nestmates an individual receives queen pheromone from). **g**-**h** Dimension reduction reveals that workers follow stereotypical developmental trajectories as they age. **g** Principal component analysis of the six behaviours shown in panels **a**-**f**. Background points represent a given individual on a given day. Foreground points show the mean for a given age cohort on a given day. Point colours represent colony identities. **h** Blowup of the rectangular area in **g**. Coloured arrows show developmental trajectories for each colony. The black arrow shows the ‘global’ trajectory. Coloured points show the inflection point for each colony. Points marked with an ‘x’ indicate the 3–5 day-old workers, which contribute most to feeding the queen and raising new queens. **i**-**k** The daily contact network (**i**-**j**) and the daily pheromone relay network (**k**) for colony 18 on 29/8/2016. All networks used the same layout, obtained by applying a force-directed layout algorithm to the contact network. Nodes represent individual bees, and the queen is indicated by the white node. Node size indicates age. In (**i-j**), edge thickness represents the number of pairwise encounters. In (**k**) edge weight & direction indicates pheromone flow. In (**i**), the nodes are coloured according to the community scores. The network is partitioned into three overlapping communities; a foraging community consisting mainly of older workers (red nodes), and two nurse communities consisting mainly of younger workers (blue and green nodes). In (**j**) nodes are coloured according to the nurse bridging score, $H^\prime (N_A,N_B)$. Bees positioned at the overlap of the two nurse communities have high bridging scores. In (**k**) nodes are coloured according to the out degree. **l** The coarse-grained flow network. Node labels indicate the age in days. Weighted and directed edges indicate the net pheromone flow between cohorts. The donation hierarchy is indicated by the vertical positions of each node, as defined by the dominance ranks

As social insect worker behaviour typically follows a noisy, but stereotyped developmental trajectory [26, 28, 48], we next tested whether the expression of behaviours associated with messaging also exhibit predictable changes with age. To capture the multi-faceted nature of messaging, we defined a suite of six measures closely tied to messaging (‘messaging syndrome’), and used dimensionality reduction to quantify the developmental trajectory of these traits. As the most straightforward definition of ‘messaging’ requires that a worker frequently encounters the queen, our first two measures were (i) the active (i.e., beyond random) attraction of individual workers towards the queen [49], and (ii) the number of encounters between each worker and the queen. Queen attraction rapidly increased over the first few days of age, peaked at 3–4 days, and gradually declined thereafter (Fig. 7a). Similarly, newly-eclosed callow workers (0-days old) had few queen contacts, but over the first few days of life the number of contacts rapidly increased, reaching a peak at 3–4 days, and steadily decreasing thereafter (Fig. 7b).

As the social networks of honeybees are modular [29, 50, 51], the topological position of a bee, and in particular, its proximity to the queen, could influence how effective it is at spreading the queen pheromone. Given that queens were always most strongly affiliated towards one of the two nurse groups identified by the community detection algorithm, our third measure quantified how workers’ combined nurse affiliations ($N_A + N_B$) varied with age. Workers under 4 days of age were entirely affiliated to the nurse groups, but from $\sim$5 days of age, they began to transition into the forager group (Fig. 7c,i), with the S-shaped form of the transition closely resembling that seen in ant workers [28].

Given that nurses that were equally affiliated to both nurse groups might be well placed to act as messengers, we next used the entropy across their two nurse scores to calculate a ‘bridging score’, quantifying the extent to which each bee was positioned at the overlap between the nurse groups ($H^\prime (N_A,N_B)$ , Fig. 7d,j, see Methods). Newly-eclosed workers had low bridging scores because they exhibited strong spatial fidelity to one of the two broodnests [14, 29]. However, over the first few days of life the bridging score rapidly increased, reaching a peak at $\sim$4–5 days, and declining thereafter. Therefore, 4–5 day old workers are best placed to convey the queen pheromone to those responsible for raising replacement queens.

As messengers should play a major role in disseminating the queen pheromone, the fifth and sixth measures quantified workers’ spreading roles over the daily pheromone relay networks (Fig. 7k, see Methods). Newly-eclosed workers donated to few nestmates (i.e. had out degree, *k*_*out*_∼100) and were net pheromone receivers (i.e., $\Delta k < 0$, Fig. 7e-f). However, over the first few days of their life the number of nestmates to which they donated rapidly increased, so much so that that by $\sim$3–4 days their out degree had tripled and they were now net pheromone donors ($k_{out} \sim 300$ and $\Delta k> 0$).

We next show that the expression of the six messaging behaviors follow a stereotyped developmental trajectory, and that the period of maximum developmental change coincides with a key point in the life of a worker. To do so we perfomed principal components analysis on the six metrics described above. Together the first and second principal components (PC1 & PC2) explained 58% of the variance, however, a scatterplot of the first and second components revealed little structure (Fig. 7g). Therefore, to investigate whether worker messaging follows a stereotyped developmental progression, we calculated the mean PC1 and PC2 for each day of age. This revealed a C-shaped cluster of points (Fig. 7g), and trajectory smoothing confirmed that newly-eclosed workers started at one end of the C, and progressed toward the other as they matured (Fig. 7h). Interestingly, the trajectories of all colonies contained a sharp inflection point at 3.8±0.3 days of age (grand mean ± standard error; Fig. 7h, solid points), which exactly coincides with the age at which bees contribute most to two key nurse-associated tasks, namely feeding the queen [20] and the brood [14, 40–42] (Fig. 7h, crosses).

To more formally investigate the organisation of donor-receiver relations between workers of different ages, we combined the daily relay networks into a single coarse-grained network in which the nodes represent age groups, and edges represent the net pheromone flow from one age cohort to another (Fig. 7l). As the donor-receiver relationships that constitute the coarse-grained relay network are similar to winner-loser relationships in dominance hierarchies, we used a standard dominance score [52] to rank the age cohorts according to their position whithin the donation hierarchy. This revealed that the donation hierarchy is closely associated with age: the 3–5 day-old workers occupied the top position within hierarchy, indicating that they acted as the most prominent net donors to all other age groups. Older cohorts occupied ever lower positions as age increased, reflecting a decreasing importance in relaying information about the queen’s presence with worker age. The only exception to this was the 0–2 day old callows, which occupied an intermediate position in the hierarchy. Taken together, our results show that the $\sim$3–5 day-old bees are key distribution hubs in the pheromone relay networks, which is a key expectation if they act as messengers.

## Discussion

The means by which a laying honeybee queen communicates her continuing presence to a colony of tens of thousands of sterile workers has been a focus of much study [5, 7, 13–16, 19–25, 39, 53], yet there has been little recent progress in quantifying the communication channels that allow the queen pheromone to spread throughout the hive. In this paper we used automated behavioural tracking and an empirically-parameterised simulation of pheromone transmission over the time-ordered sequence of physical bee-to-bee encounters, to quantify both direct and indirect transmission of queen pheromone throughout the colony. Consistent with previous work [14], we found that queens alternate between stationary and travelling states. Furthermore, we also found that each state is associated with a distinct transmission regime: whilst stationary queens exposed a relatively small worker audience to large quantities of queen pheromone, travelling queens exposed a larger audience to smaller quantities. As well as confirming that queen behaviour directly influences the size of the queen’s ‘audience’ [14], our results are consistent with the suggestion that the queens’ frequent alternation between states helps balance a trade-off between the size of the audience and the exposure of each audience member.

Given that physical encounters between the queen and inanimate objects can induce workers to orient towards the object [5, 13], bees that have recently participated in the retinue – where they are able to make intimate and prolonged contact with the queen – have long been suspected of acting as ‘messengers’ of the queen’s fertility signal [13–15]. Fine-grained analysis of the spatial trajectories of individual workers confirmed that the presence of the queen is communicated by indirect ‘messaging’, that is by worker-to-worker encounters, as bees oriented more strongly towards post- than pre-retinue nestmates; and because bees tended to orient more strongly towards post-retinue nestmates that were carrying high pheromone loads than towards low-load nestmates. Simulations indicated that this indirect communication plays a critical role in ensuring that a large portion of the colony, especially the nurses, are informed of the presence of the queen. Furthermore, contrasting the behaviour of pre- versus post-retinue bees showed that workers display active behavioural changes, as the encounter with the queen was followed by increases in worker mobility, which have been suggested to assist in the dispersal of the queen pheromone throughout the hive [14, 23]. Comparisons between the speed of queen pheromone transmission over the bee-to-bee contact sequences immediately before versus immediately after a queen encounter confirmed that transmission was faster after the encounter. Such differences could only exist if the bee-to-bee contacts from the periods following encounters with the queen include causal sequences that promote spreading, such as contact cascades initiated by excited messengers.

Leveraging the wide demographic range covered by our age-marking scheme, we found that worker bees exhibited characteristic changes in a suite of messaging-related behaviours as they aged. Over the first few days of their lives, workers exhibited major changes in their attraction toward the queen and an associated rapid increase in their queen encounters. During this period workers underwent a major transition across the colony social network, arriving at positions that were central (i.e., close to the queen) and that were ‘bridges’ between groups of spatially isolated nurse workers. The transmission model also confirmed that these changes were also associated with a corresponding increase in the extent to which workers acted as donors of the queen pheromone. Remarkably, this brief period coincides with the age when workers are most responsible for the critical tasks of feeding the current queen [20], and in the event of her death, for raising a replacement queen by provisioning newly-eclosed larvae with royal jelly [40–42]. Indeed, workers’ hypopharyngeal glands – which secrete the royal jelly – are already well-developed by 5 days [54], and royal jelly production enters its peak at 6 days [55]. These coincidences suggest that messaging may represent one task within a wider queen-focused role, performed mainly by the 3–5 day old bridging nurses. This arrangement also makes sense from an ergonomic perspective, as the individuals with the most intimate associations with the queen are also best-placed to quickly detect and react to her death or waning fecundity. To formally test whether the 3–5 day old bridging nurses fulfil such a role, it would be necessary to compare colonies that have lost both their queen and their messengers, versus colonies that have lost both their queen and a random selection of same-age workers.

## Conclusions

More broadly, honeybee communication is of particular importance for our understanding of language, and what sets human language apart from non-human animal communication. Displacement communication – the ability to refer to things that are spatially and temporally remote – is regarded as one of the defining features of human language [56–58]. The most striking equivalent in non-human animal communication is the ‘dance language’ of the honeybee [59], in which returning foragers signal the quality and spatial location of a food source through stereotyped movements, which are closely followed by an audience of receivers [60]. Our results confirm an additional channel of displacement communication in the honeybee – messenger workers actively change their behaviour following an encounter with the queen to signal her continuing presence to nestmates that are spatially or temporally remote from the queen.

## Methods

### Observation colonies

Ten queenright honeybee colonies (subspecies *Apis mellifera carnica*) were kept in the campus apiary of the University of Lausanne. Colonies were housed in modified observation nests, consisting of a single continuous 64$\times$44 cm wax comb containing honey, pollen, and developing brood on either side [26] and sandwiched between two glass walls [26]. To ensure colonies were exposed to diurnal temperature and light cycles, each observation nest was placed inside an exterior sheltered housing. These housings consisted of a large rectangular box (1$\times$1$\times$2.5 m), with two tracking cameras placed at opposite ends of the box for recording the movements of bees on either side of the wax comb through the glass walls. To reproduce the natural inside-nest conditions, the housings were closed to prevent the incursion of natural light. A hollow tube connecting the observation nest to the side-wall of the housing allowed bees to come and go at will.

### Barcode tagging

Small paper tags (1.86$\times$1.86 mm) bearing a unique two-dimensional barcode (AprilTag, 36h10 family [61]) were attached to the thorax of each worker to allow automated tracking. Tags were glued to the dorsal thorax using a small drop of adhesive (Pattex Power Easy Gel) after the individual had been temporarily immobilized by chilling on ice.

### Generating cohorts of known-age workers

Data on individual worker ages were acquired by introducing successive cohorts of tagged callow workers of known age [26]. A cohort of callow tagged workers was produced by removing a frame containing mature brood from the broodnest of a donor colony, and incubating this frame overnight at 34.5°C. The next day, 290 newly-eclosed workers were harvested from the frame, and an AprilTag barcode affixed to each. The tagged cohort was then immediately introduced into the observation nest. Each colony was subjected to eight cohort introductions, with a three-day interval between successive introductions. To minimise rejections of newly-tagged bees, the callows were introduced through access-holes in the top of the nest. As the callows sometimes took several hours to fully integrate with the rest of the colony, we did not analyse the tracking data from cohort introduction days. In order to obtain behavioural records of a wide range of different-aged workers, we analysed seven days of tracking data [62], covering three days before and four days after the introduction of the eighth cohort. A subset of this dataset (three out of seven days) were analysed in a previous study [29].

### Automatic tracking

High resolution digital video cameras operating at two frames per second were used to identify the location and orientation of each tag across successive images [30]. All colonies were continuously tracked from the introduction of the first cohort until five days after the eighth cohort. We used an established software pipeline [30, 37, 63] to extract the spatial trajectories of each worker, and to identify and remove cases when a tag was identified in a location where there was no tag (false positives), or when the identities of two tags were exchanged (tag-confusions).

### Identifying physical contacts between bees

To identify transmission-relevant contacts between bees we used a geometric approach in which the body outline of each bee is modelled as a trapezium, centered on the tag [30]. The trapezium lengths were scaled to approximate the differing body lengths of workers and queens. Furthermore, to reflect the broad reach of the antennae, they were wider at the head-end than at the tail-end. As the vast majority of queen pheromone transfer occurs when one bee inspects (i.e., licks or antennates) the body surface of a nestmate [16], we defined a contact as any instance in which the trapezium head-end of one bee overlapped with any part of the trapezium of a nestmate. This specification ensured that physical contacts not relevant for transmission, such as when bees were standing side-by-side, or tail-to-tail, were excluded.

### A simulation model for queen pheromone transmission

As the queen pheromone is only produced by mated queens, and because honeybee colonies have only one queen, in our model the queen was the only source of *de novo* pheromone. The load carried on the queen’s cuticle was assumed to be constant, reflecting the equilibrium between production and removal by the retinue workers [16]. By contrast, workers were modelled as queen pheromone sinks, with zero load at the start of each simulation, and able to acquire pheromone either directly by contacts with the queen, or indirectly by contacts with pheromone-carrying workers. The load carried on the cuticle of a worker experienced a constant exponential decay [15, 39], reflecting absorption into the cuticule and deposition onto the comb.

The spread of the queen pheromone was modelled as a diffusive process operating on the time-ordered sequence of physical encounters between workers. As the direction of transmission during a given encounter depends on which individual licks or antennates the other [16], we used the tag orientations to obtain the heading of both participants, *i* & *j*, in each encounter. These headings were then used to classify each individual as either ‘inspecting’ (receiver) or ‘being inspected’ (donor). For example, when *i* faced toward *j* and *j* faced away from *i* then individual *i* was inspecting *j*, and hence there was transmission from *j* to *i*. When neither *i* nor *j* faced towards the other, there was no inspecting, and hence no transmission. When both *i* and *j* faced towards each other, they were both inspecting, in which case there was transmission from the bee with the higher load to the bee with the lower load.

The loads of the donor and receiver were updated dynamically throughout the contact, and the model was parameterised to reproduce the previously-reported association between contact duration and the volume transferred [16]. This was achieved by first estimating the proportion of the contact spent licking rather than antennating the donor based on the total contact duration [5] (Additional File 1: Fig. S4), and then applying different transfer rates for licking and antennating [16].

The model also included a term to reflect the diminishing returns associated with longer encounters that a receiver might experience in obtaining pheromone from a donor (Additional File 1: Fig. S5), for example because the more it removes from the body surface of the donor, the greater the difficulty in obtaining more [16]. Finally, to reflect the previously-reported observation that transmission is ‘leaky’ (receivers ingest approximately half of the load obtained during an encounter, and hence the ingested portion becomes unavailable for onwards transmission to other nestmates [16]), the acquisition rate of the receiver was set at half the loss rate of the donor.

The parameters governing queen pheromone production, transfer, and decay rates were taken from previous experimental studies [5, 16, 39]. Thus, workers were considered to be informed of the queen’s presence if their simulated pheromone load at a given time, $\lambda (t)$, was greater than the previously-established worker sensitivity threshold to queen pheromone $\lambda _{min}$ = 9.03 pg [16, 38]. Receivers were labelled ‘directly-informed’ when their load was above the sensitivity threshold $\lambda _{min}$ and they had obtained more pheromone from the queen than from other workers (i.e., bees with $\lambda (t)> \lambda _{min}$ & $\lambda (t)_{q \rightarrow w}> \lambda (t)_{w \rightarrow w}$). Receivers were labelled ‘indirectly-informed’ if their load was above the sensitivity threshold and they had obtained more pheromone from other workers than from the queen (i.e., bees with $\lambda (t)> \lambda _{min}$ & $\lambda (t)_{w \rightarrow w}> \lambda (t)_{q \rightarrow w}$).

### Measuring transmission dynamics

The dynamics of queen pheromone transmission were quantified using three metrics. First, we measured the ‘audience size’ A(*t*), that is, the proportion of the population of tagged bees that were in the informed state. Second, we measured the ‘audience coverage’ S(*t*), that is, the mean of the Euclidian distances between all unique pairs of informed bees. In cases in which informed bees appeared on the other side of the comb, distances between pairs of bees on different sides of the comb were calculated using shortest straight-line distance around the sides of the frame. Third, we measured the mean quantity of queen pheromone carried by the informed bees, that is, the ‘audience load’ L(*t*).

### Transmission within queen bouts

Disentangling the effect of queen state on transmission was complicated by rapid switching between bouts of Stationary or Traveling behaviour (Fig. 1a-b), such that the transmission chains originating from a given bout merge into chains from the preceding and succeeding bouts. To circumvent this issue, we considered each bout independently, assuming all workers were uninformed (i.e., had zero load) at the start of a bout, and simulated queen pheromone transmission over the physical encounters that occurred within that bout (Fig. 1d-e). As the transmission model was stochastic, repeat runs could produce different transmission chains, hence the model was applied 100 times to each bout and the three audience size measures averaged across the 100 runs.

Queen bouts that were very short (< 10 seconds), or that had low classification confidence (HMM state probability, *p*<0.75) were excluded from this analysis. However, because most bouts were short (grand mean ± standard error: 55±7.8 s, n=10), and because the bout duration distribution was highly right-skewed (grand mean ± standard error of skewness: 5.4±0.4, n=10, Additional File 1: Fig. S6), not all 10 colonies were represented in at least one of the observation days at bout durations above $t=$ 305 seconds. Therefore, we applied an upper cutoff of 305 seconds to the within-bout simulations.

### Transmission over the daily contact sequence

To quantify the role of each worker in spreading the queen pheromone during a given day, we ran the transmisison model over the temporal sequence of physical encounters that started and finished between 00:00:00 and 23:59:00 on that day. As for the within-bout simulations, the workers were initiated with zero loads, and 100 model runs were applied to each 24-hour contact sequence.

### Rayleigh test of bees’ orientations toward post-retinue nestmates

To quantify how workers react to a nestmate that has recently encountered the queen, we identified all occasions when bees were within two body-lengths of a post-retinue bee, and then rotated their corresponding trajectory sections into positions and headings relative to the position and heading of the post-retinue bee. As there was an a-priori reason to expect that bees should orient their bodies to face toward a post-retinue bee, we used the variant of the Rayleigh test in which the alternative hypothesis is a ‘specified direction’, for which we supplied the heading from the position of the receiver, *i*, to the nearest body-part of the post-retinue bee $\mu$ (Additional File 1: Fig. S7). In this variant of the Rayleigh test, the test statistic, $\rho$, measures the agreement between the observed distribution of receivers’ body orientations, and the specified direction to the post-retinue bee,

$$\begin{aligned} \rho = \frac{\sum _{i=1}^{n} cos(\theta -\mu )}{n} \end{aligned}$$

where $\theta$ represents the observed receiver body orientations relative to the post-retinue bee, $\mu$ is the specified direction, and *n* is the sample size. Thus, $\rho$ is bounded in the range (−1,1), and approaches 1 when the receivers face toward the body of the post-retinue bee (i.e., when $\theta \sim \mu$), and approaches −1 when they face away from the post-retinue bee (i.e., when $(\theta - \mu ) \sim \pi$). Importantly, $\rho$ is close to zero when $\theta$ is uniformly distributed, or when the mode of $\theta$ is orthogonal to $\mu$ (i.e., when $(\theta - \mu ) \sim \pi /2$). All Rayleigh test calculations were carried out using the *rayleigh.test* function from the *circular* library [64] for *R*. For a description of the procedures for calculating the difference maps for $\rho$, see the Supplementaty Information.

### Worker attraction to the queen

To test for attraction of workers to the queen we used the approach of [49]. This method compares the observed distances between simultaneously-observed positions of two individuals A and B, with the distances between all pairs of points in the trajectories of both individuals, irrespective of whether they were simultaneously observed or not. Following [49], to establish whether a given worker exhibited statistically significant attraction towards the (stationary) queen, we performed a paired Wilcox test comparing the observed and expected distances. As we had an a-priori expectation of attraction, we performed a one-tailed test with the alternative hypothesis that the observed distances were shorter than the expected distances.

Whilst this approach allows attraction of A to B (or of B to A) to be distinguished from mutual attraction of both A and B to a shared spatial location, it cannot distinguish between attraction of individual A to individual B, or of B to A, when both A and B are simultaneously moving. Therefore, to ensure that our results quantify the attraction of workers toward the queen, not the attraction of the queen toward particular workers, we only considered times when the queen was in the stationary state.

### Constructing daily relay networks

The time-ordered sequence of pheromone transfers produced by the daily transmission simulations were used to construct a time-aggregated relay network describing the flows of queen pheromone between all unique bee pairs in a colony during a 24-hour period. The nodes in this network were linked by weighted and directed edges, representing the summed queen pheromone flow from worker *i* to *j* that is,

$$\begin{aligned} Q_{i \rightarrow j} = \frac{\sum _{s=1}^{S} \sum _{n=1}^{N_s} {q_n}_{i \rightarrow j}}{S} \end{aligned}$$

where ${q_n}_{i \rightarrow j}$ indicates the volume of queen pheromone transferred during the $n^{th}$ of $N_s$ queen pheromone transfers from *i* to *j* during simulation *s*, and *S* representes the total number of simulations ran ($S=100$). The summed queen pheromone flow in the opposite direction, $Q_{j \rightarrow i}$, was calculated in the same way, hence each pair of nodes could be connected by up to two edges.

To assess the role that each individual plays in disseminating the queen pheromone, for each bee we calculated, (i) the number of nestmates to which it donated queen pheromone (i.e., its out-degree $k_{out}$), and (ii) the difference between its out- and in-degree ($\Delta k = k_{out} - k_{in}$).

### Soft community detection on daily contact networks

Each daily encounter network represented a 24-hour period (0:00:00 to 23:59:59), and was constructed by counting the number of physical encounters between each pair of tagged individuals in the colony during that period. To detect communities in the daily encounter networks we used an open-source implementation [65] of the FacetNet soft community detection algorithm [28, 43, 66, 67]. Facetnet uses a continuous score (in the range 0–1) to quantify the affiliation strength of a given network node to a given community, subject to the constraint that, given N communities, the N scores for a given node sum to 1. Thus, a bee that is strongly affiliated to the forager community, but weakly affiliated to the two nurse communities might score {*F*=0.8, $N_A$=0.1, $N_B$=0.1}. For details on the identification of the optimal number of communities, and how they were labelled, see the Supplementary Information (Additional File 1: Fig. S3).

### Identifying bridging nurses

We used information-theoretic measures to quantify the extent to which an individual occupies the overlap between the two nurse communities, that is, the ‘nurse bridging score’,

$$\begin{aligned} H^\prime (N_A,N_B) = H(N_A,N_B) / H_{max} \end{aligned}$$

where $H(N_A,N_B)$ represents the entropy of the FacetNet scores for the two nurse communities, and $H_{max}$ represents the maximum possible entropy, that is, for an individual that exhibits identical affiliations to both nurse communities. Thus, $H^\prime (N_A,N_B)$ is bounded in the range 0–1, and bees that are equally affiliated to both nurse communities (e.g., when $N_A{=}^{\ 1\!}/_{3}, N_B{=}^{\ 1\!}/_{3}, F=0$) will have $H^\prime (N_A,N_B)=1$, whereas bees that are associated with only one nurse community (e.g., when $N_A=1, N_B=0, F=0$) will have $H^\prime (N_A,N_B)=0$.

### Principal components analysis of worker messaging

The individual-level data descibing the six ‘messaging’ metrics exhibits considerable uncontrolled variation both between colonies (e.g. due to differences in numbers of tagged individuals), and across days within a colony (e.g. due to changes in temperature and rainfall). To prevent this variation from introducing heterogeneities in the PCA (and hence obsucre the characterisation of the developmental trajectories), we subjected each of the six metrics to a quantile transformation. Transformations were carried out independently for each of the six metrics, and for each of the 70 observation days (10 colonies, 7 days each). These within-day quantiles were combined, and a single PCA conducted on the pooled data.

To characterise the developmental trajectory of the workers in each colony, we used the means of the first and second principal components for workers of all 0–24 days (Fig. 7h), and then subjected these means to trajectory smoothing, using the function *TrajSmoothSG* from the *trajr* library for *R* [68].

## Supplementary Information

Additional file 1: A document containing supplementary methods and results accompanies the paper.

## Data Availability

The tracking dataset, and the code for the transmission model are available in the Zenodo repository, https://doi.org/10.5281/zenodo.8192185.

## Abbreviations

HMM: Hidden Markov model
LMM: Linear mixed model
GAMM: Generalised additive mixed model
PCA: Principal components analysis
