# Supplementary material for: Pheromone relay networks in the honeybee: messenger workers distribute the queen’s fertility signal throughout the hive
Source: BMC Biol. 2024 Dec 18;22:288. doi: 10.1186/s12915-024-02083-w (PMC11658054; doi:10.1186/s12915-024-02083-w)
Supplement: Supplementary file 1 — Additional file 1: A document containing supplementary methods and results accompanies the paper. [file 12915_2024_2083_MOESM1_ESM.pdf]

Supplementary Information

Pheromone relay networks in the honeybee: messenger workers distribute the queen's fertility signal throughout the hive

Thomas O. Richardson, Tomas Kay, Laurent Keller, and Nathalie Stroeymeyt

Dataset summary

The honeybee data analysed here originate from an experiment first described in Richardson *et al.*, 2022 [29]. Whereas that study investigated the three days following the introduction of the eighth cohort, we here expand this period to include the day of the 8th cohort, and the three days immediately before and after it [62]. Extending the study period from 3 to 7 days increased the size of the dataset from 9.7×10^8 to 2.1×10^9 individual trajectory coordinates (Table S1).

Colony	Colony size (est.)	N tagged bees	N trajectory fixes	Total bee hours
1	3656	768	210 417 744	29 225
2	4676	759	286 141 127	39 742
5	4127	738	201 581 258	27 997
6	3673	793	241 078 887	33 483
9	4813	1531	213 721 061	29 683
10	4851	1261	143 537 639	19 936
12	3527	1288	341 151 386	47 382
16	6291	1217	169 081 050	23 483
17	4851	1267	135 617 714	18 836
18	2867	1271	199 942 358	27 770
Σ	43 332	10 893	2 142 270 224	297 537

Table S1: **Colony-level data summary.** Colony sizes were estimated on the day of the introduction of the 8th cohort by quadrat sampling. The number of tagged bees represents the count of the unique tagged bees in the colony over the seven observation days. The number of trajectory fixes is the total number of tag positions observed across the seven days.

Queen state detection

To identify periods of queen activity, we used the *moveHMM* package [34] for *R* to model queen movement as a Hidden Markov Model (HMM). To do so we extracted the sequences of frame-to-frame step-lengths and turn angles from the trajectory of each queen. The step length distributions were highly right-skewed, and contained many zero-distance steps, hence a zero-inflated Gamma distribution was used to model the steps in the HMM. As the empirical distributions of turn angles resembled a normal distribution centered on 0, a zero-centered Von Mises circular distribution was used in the HMM.

As the HMM fitting procedure involves stochastic optimisation, repeating the procedure on the same trajectory typically results in a number of HMMs each with different parameters describing the two states. Therefore, to improve the quality of the chosen HMM we use a two-stage process in which we first fitted 100 HMMs to each daily trajectory, and then calculated the average parameter values aross the 100 intitial models as starting points for the final fit.

Application of the Viterbi algorithm to the 70 daily queen trajectories identified 333,637 bouts of stationary (S) or travelling (T) behaviour, with a mean per-bout state probability of 0.92±0.0002. Furthermore, the small standard errors associated with the parameters used to fit the distributions of

step lengths and turn angles in each HMM (Table S2) indicated that the two states were consistent across queens. The average duration of a stationary bout was longer than the average duration of a travelling bout, with all 10 queens spending a slightly greater proportion of their time in the stationary state (mean time in state, S; 58%, n=10) (Table S2).

State	Step lengths			Turn angles	Bout	Bout
	mean, μ	standard deviation, σ	zero mass	concentration, κ	probability	duration (s)
T	0.067±0.0049	0.00032±0.0002	0.0096±0.0021	0.88±0.06	0.92±0.01	21±5.2
S	0.0075±0.00036	0.000014±0.00002	0.41±0.038	0.4±0.074	0.89±0.011	30±8.2

Table S2: **Summary of the step length and turn-angle distributions fitted by the hidden Markov models to the daily queen trajectories.** All values are grand means and standard errors, calculated using the 10 means for the individual queens. ‘S’; stationary state. ‘T’; travelling state. The step length distributions were modelled using a gamma distribution, and the turn angle distributions were modelled using a zero-centered Von Mises circular distribution. State probabilities were inferred using the Viterbi algorithm.

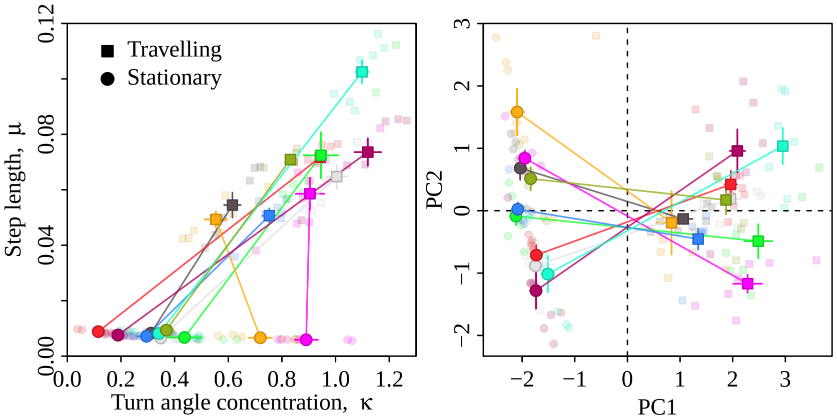

Figure S1: **Summary of two-state Hidden Markov model for queen movement.** (**a**) The within-bout step length as a function of the within-bout turn angle concentration parameter. Point colours indicate colony identity. Small points indicate the daily means for a single queen, averaged across all bouts. Large points and lines indicate the within-colony grand means and associated standard errors, averged across the daily means. Lines connect the within-colony grand means. (**b**) Principal components decomposition of the step length, step length standard deviation, step length zero mass, turn angle concentration, bout probability, and bout duration. The two states are well separated.

Queen-worker encounter kinetics

As the mobility of the honeybee queen has been suggested to play a critical role in the transmission of the queen pheromone and the rearing of new queens [5,14,18], we investigated how the queen's behavioural state influenced her physical encounters with workers. The main paper presented the statistical analyses showing that honeybee queen encoutners are consistenty with the predictions of standard encounter kinetics [35], that is, travelling queens experienced a significantly higher encounter rate than stationary queens. Figure S2 provides a visual representation of the data on which those statstical analyses were based.

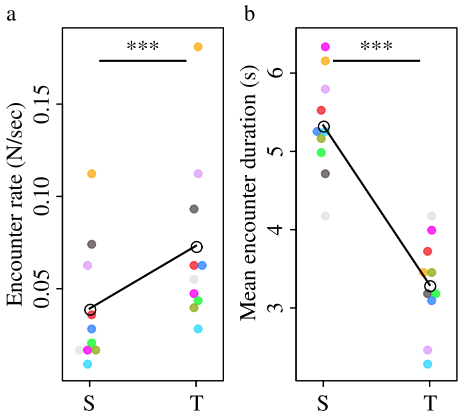

Figure S2: **The behavioural state of the queen influences encounter characteristics.** S: queens in the stationary state. T: queens in the travelling state. Coloured points represent colony means, averaged across the 7 days. Black points indicate grand means, averaged across the 10 colony means. Asterisks indicate the results of the LMMs described in the text (***; $p < 0.0001$, n.s; $p > 0.05$).

Community detection and labelling on daily contact networks

To identify the optimum number of communities in the daily encounter networks, we measured the soft modularity [43] associated with FacetNet partitions from 2 to 5 communities. Across all 10 colonies, the soft modularity exhibited a peak at 3 communities (Fig. S3), so for all analyses we used the FacetNet partition associated with 3 communities.

The FacetNet algorithm provides arbitrary labels to differentiate the communities. Therefore, following several previous studies [28,29,30,66,67], we used several spatial and demographic features of the unlabelled communities to assign them to biologically meaningful categories . Social insect colonies are characterised by a basic distinction between young workers at the core of the nest (nurses) and older, more peripheral workers (foragers) [26,27], we defined five metrics expected to be tightly correlated with the nurse-forager axis, namely;

1. Age (older for foragers)
2. Number of side switches (more for foragers)
3. Mean distance to the nest entrance (shorter for foragers)
4. Mean distance to the nest interior walls (shorter for foragers)
5. Proportion of time spent inside the broodnest (less for foragers)

The rank-order of each community within each metric was then obtained, and the five ranks of each community summed. Thus, a community whose members were the oldest, switched sides the most, were closest to the nest entrance and to the nest walls, and that spent least time in the broodnests, achieved a summed rank of 15. The community with the highest summed rank was then labelled the 'Forager' community. As the members of the remaining two communities typically spent a large amount of time inside one or both of the two broodnests, we labelled these 'Nurse A' and 'Nurse B' communities. After labelling, the FacetNet community scores were used as a quantitative indicator of the affiliations of each individual to the forager (F), Nurse A (N_A) and Nurse B (N_B) communities.

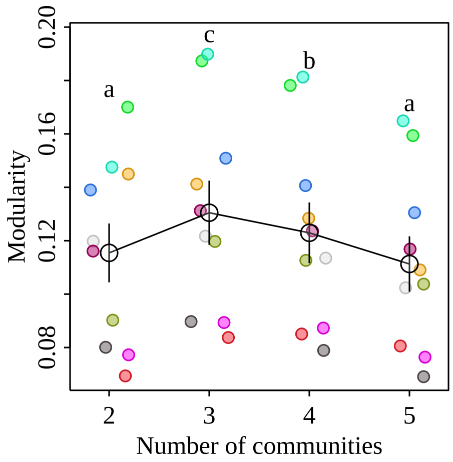

Figure S3: **Honeybee contact networks consist of three partially-overlapping communities.** The y-axis shows the 'soft' modularity as a function of the number of communities detected by the Facetnet algorithm. Colours represent colony identities. Each point represents the mean modularity for a given colony, with the mean calculated across the 7 daily networks spanning a three-day period before and after the intridiuction of the 8th cohort of - day-old tagged callow workers. White points the grand means calculated across the 10 colony means, and the bars give the standard errors thereof. Lower-case letters indicate Tukey post-hoc contrasts applied to a linear mixed model in which the response was modularity and the predictor was the number of communities, and where colony identity was a random effect ($\chi^2 = 57$, d.f. = 1, p < 0.0001).

Although the community affiliations of the queen were not used to help label the communities, in all 70 daily networks the queen always exhibited stronger ties to the nurse communities than to the forager community.

A simulation model for queen signal transmission

To simulate the transmission of queen pheromones over the time-ordered contact sequences, we developed a temporally-explicit stochastic model inspired from standard temporal Susceptible-Infectious epidemiological models and parameterised using published experimental data (see below).

In our model, the queen was the only primary source of *de novo* pheromone. The amount of pheromone carried by the queen was considered to be at equilibrium [16] and had a constant value of λ_{queen} throughout the simulations. By contrast, workers could acquire pheromone either directly from the queen or indirectly from other workers, and pheromone carried by workers experienced a constant exponential decay with rate k. At the onset of each simulation, the queen was initialized with load λ_{queen} whilst all workers were initialized with a zero load.

We then considered all tracking frames sequentially and for each frame n, we performed the following steps in order.

(i) Pheromone decay

First, the loads of all pheromone-carrying worker bees were updated to account for decay according to the following formula:

$$\lambda_{i,n} = \lambda_{i,n-1} \cdot e^{-k(t_n - t_{n-1})},$$

where $\lambda_{i,n}$ is the load of worker i at tracking frame n occurring at time t_{n}, and $\lambda_{i,n-1}$ is the load of worker i at the previous frame $n-1$ occurring at time t_{n-1}, and k is the decay rate. The parts of each worker's load originating directly from the queen or indirectly from workers were also updated to account for decay using the same approach.

(ii) Identifying all pairs of bees in contact and initialising new contact pairs

Using the list of sequential contacts extracted from the tracking data, we identified all pairs of bees that were in physical contact at frame n, that is, all pairs of bees who had a contact starting at or before frame n and ending at or after frame n.

If frame n was the first frame of a focal contact, we determined which bee of the pair was the potential donor and which was the potential receiver. As pheromone transfer typically occurs via the receiver licking or antennating the donor, we used the geometric configuration of the two bees to identify the potential donor and receiver. More specifically, if only one of the two bees' heads overlapped with the other's body, then the former was considered as the potential receiver and the latter as the potential donor. By contrast, if both bees' heads overlapped with the other's body, then the bee with the higher load was considered as the potential donor.

As licking is associated with a pheromone transfer efficiency several orders of magnitude greater than antennating [16], it was necessary to estimate the proportion of time that the receiver bee spent licking (rather than antennating) the donor, to obtain a more accurate estimate of the amount of pheromone transferred during the focal contact. To do so, we used previous empirical data showing a tight positive association between the duration of the contact and the proportion of time spent licking [5]. Therefore, if frame n was the first frame of the focal contact, we also calculated the proportion p_{lick} of time spent licking as a function of the total duration of the focal contact, using the following equation (see 'Model parameterisation' section below for details):

$$p_{lick} = \beta + (1 - \beta) \cdot (1 - e^{-\frac{\Delta t}{\tau}})$$

where p_{lick} is the proportion of time spent licking during the focal contact, β is the probability of licking for instantaneous (zero-duration) contacts, Δt is the duration of the focal contact, and τ is the half-life constant ($\tau = \frac{(1-\beta)}{2}$).

Finally, if frame n was the first frame of the focal contact, the donor load available for transfer, λ'_{donor}, was considered to be equal to the whole donor load, λ_{donor}, at frame n (see below).

(iii) Determining the amount of pheromone transferred for each pair of bees in contact

We then iterated over all pairs of bees that were in contact at frame n. For each focal pair of bees, we first determined whether any pheromone was transferred at frame n. Transfer occurred if the two following conditions were met: (i) the potential receiver was not the queen; and (ii) the available load λ'_{donor} of the potential donor at frame n was higher than the load threshold λ_{min}.

If both conditions were met, we next determined whether frame n corresponded to licking or antennating for the focal pair of bees. This was determined via a uniform stochastic processed with a probability of licking of p_{lick}, determined when initialising the focal contact as explained above.

We then calculated and recorded the amount of pheromone $\lambda_{transferred}$ transferred from the donor to the receiver at frame n using the following formula:

$$\lambda_{transferred} = \gamma_{type} \cdot \lambda'_{donor}$$

where γ_{type} is the per-frame transfer rate for either licking or antennating frame (see below for parameterisation), and λ'_{donor} is the donor load available for transfer at frame n.

(iv) Updating the loads of donor and receiver bee

Once the amount of pheromone transferred from the donor to the receiver was determined, the loads of both donor and receiver bees were updated as follows:

$$\lambda_{donor} = \lambda_{donor} - \lambda_{transferred} \text{ (except if donor = queen) ;}$$
$$\lambda'_{donor} = \lambda'_{donor} - \lambda_{transferred} \text{ (except if donor = queen);}$$
$$\lambda_{receiver} = \lambda_{receiver} + \lambda_{transferred} \cdot (1 - p_{ingested})$$

where $p_{ingested}$ is the proportion of queen pheromone directly ingested by the receiver upon contact, which is never available for transfer to other individuals (see below). If the donor was the queen, then

its total load λ and its load available for transfer λ' remained unchanged and equal to λ_{queen}.

Furthermore, depending on whether the donor was the queen or a worker, we calculated the total amount of pheromone carried by the receiver at frame n which originated directly from the queen or indirectly from a worker as follows:

$$\lambda_{receiver,queen} = \lambda_{receiver,queen} + \lambda_{transferred} \cdot (1 - p_{ingested}) \text{ if donor = queen;}$$
$$\lambda_{receiver,worker} = \lambda_{receiver,worker} + \lambda_{transferred} \cdot (1 - p_{ingested}) \text{ if donor = worker}$$

Finally, if the focal contact at frame n was labelled as licking, then the donor load available for transfer was further updated as follows, to reflect the observation that during a prolonged contact the pheromone is becoming increasingly difficult to acquire by the receiver (see parameterisation below):

$$\lambda'_{donor} = \lambda'_{donor} \cdot (1 - \frac{\alpha}{2i})$$

where α is an attenuation rate and i is the cumulated of licking frames since the beginning of the focal contact (see parameterisation below).

After updating the loads of the donor and receiver, the next pair of bees in contact at frame n was considered and we repeated steps (iii-iv) until all pairs of bees in contact at frame n were treated.

(v) Ending contacts

Using the list of sequential contacts extracted from the tracking data, we identified all pairs of bees who had a physical contact that ended at frame n. For all ending contacts, we recorded the total (cumulated) amount of pheromone transferred during the contact.

(vi) Recording loads

Once all contacts at frame n were processed, we recorded the load of each bee at the end of frame n, as well as what proportion of its current load originated from a direct transfer from the queen or from an indirect transfer from a worker.

We then considered the next tracking frame *n+1* and repeated steps (i-vi) until all tracking frames were processed.

Model parameterisation

- λ_{queen} = 423 ng (from Naumann *et al.*, 1991 [16]).

- The pheromone decay rate k was calculated as the sum of the decay rates associated with two complementary processes: (i) internalization of the pheromone through the cuticle (parameter $k_2 = 6.8 \times 10^{-4} s^{-1}$ in [16]) and (ii) deposition of the pheromone on the wax (parameter $k_6 = 1.31 \times 10^{-4} s^{-1}$ in [16]). This led to a total decay rate $k = k_2 + k_6 = 8.11 \times 10^{-4} s^{-1}$, which corresponds to a half-life on the worker cuticle of about 14.24 min.

- To evaluate the proportion of time spent licking (rather than antennating) during a focal contact, we used published empirical on the total length of time that individual bees spent licking or antennating the queen during any single contact (Table IIIb in Butler, 1954 [5]). As the shortest contact recorded by Butler lasted 10 seconds, whilst the shortest contacts recorded by our tracking systems lasted 0.5 second (single frame contacts), we added one additional data point to this dataset, assuming that a single frame contact is too short to allow licking and thus involves antennation only. We then used the Nonlinear Least Squares estimation (function nls from package stats in R version 4.2.1) to fit an equation of the form $p_{lick} = \beta + (1-\beta) \cdot (1 - e^{-\frac{\Delta t}{\tau}})$ to the dataset, where p_{lick} is the proportion of time spent licking during a single focal contact, β is the probability of licking for instantaneous (zero-duration) contacts, Δt is the duration of the focal contact, and τ is the half-life constant ($\tau = \frac{(1-\beta)}{2}$). The best fit had parameter values β = 0.249 and τ = 88.76 seconds (Fig. S4). Using these parameter values and the equation above, it was possible to infer p_{lick} for any focal contact in our dataset based on its duration Δt.

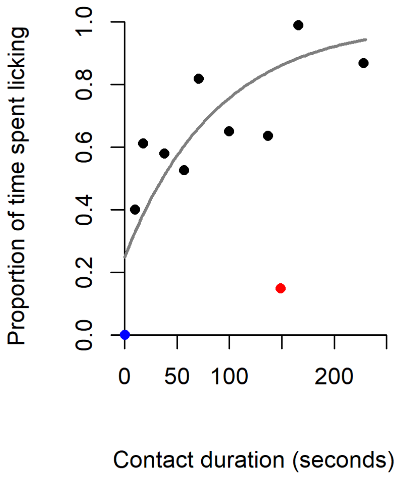

Figure S4: **Model parameterisation: relationship between contact duration and time spent licking.** Black points: data from Butler (1954) used in parameterisation; red point: outlier in data from Butler (1954), not used in the parameterisation; blue point: extra data point for single-frame contact; grey line: line of best fit with parameter values $\beta = 0.249$ and $\tau = 88.76$ seconds.

- λ_{min}, the minimum donor load for which a transfer occurred, was defined as the bees' sensitivity threshold for the queen pheromone as determined by Kaminski *et al.*, (1990) [38], i.e.:

$$\lambda_{\text{min}} = 10^{-7} \cdot 1 \ Qeq$$

  where 1 Qeq is one 'queen equivalent', that is, the average content of the mandibular gland of one mated honeybee queen. Following [16], we used a value of $1\text{Qeq} = 90.3\mu g$.

- $p_{ingested} = 0.5$ according to [16]

- To parameterise the per-frame transmission rate for licking and antennating contacts, we used empirical data provided by [16] for the pheromone load carried by receiver bees after prolonged contacts of 5s, 30s and 60 s with a dead worker bee to which 250ng of traceable (tritiated) queen pheromone had been applied (Figure 4 in [16]), and after 5-second antennation-only contacts with a lure to which 8.5ng of traceable (tritiated) queen pheromone had been applied. To be consistent with our tracking data and simulations, all contact durations were first converted from seconds to frames using our tracking frame rate of 2 frames per second (hence a five-second contact was considered to last 10 frames). In a first parameterisation attempt, we considered a constant per-frame rate of transfer for antennation and licking. Using the 5-second antennation-only empirical contact data led to an estimated per-frame rate of transfer for antennation of 1.765 frame^{-1}. We then calculated the expected proportion of time spent licking vs. antennating for each contact duration in the prolonged contact empirical dataset (5s, 30s, 60s) using the equation and parameter values described above. Using the expected proportion of time spent licking in 5-second prolonged contacts and a per-frame rate of transfer for antennation of $\gamma_{antennation} = 1.765 \times 10^{-5}$ frame^{-1}, we estimated a per-frame rate of transfer for licking $\gamma_{licking=}$0.0125 frame^{-1}. However, these estimates led to an increasingly large over-estimation of the receiver loads for longer contacts, suggesting that the queen pheromone becomes increasingly hard to acquire by the receiver during prolonged contact, e.g. because licking quickly exhausts the amount of easily accessible pheromone at the point of contact between the two bees. We therefore assumed that the amount of pheromone available for transfer on the source declined

at an increasing rate over time during a single prolonged contact, according to the following formula:

$$\lambda'_{\text{source}} = \lambda'_{\text{source}} \cdot \left(1 - \frac{\alpha}{2i}\right)$$

where λ'_{source} is the load of the source at the end of a licking frame, and i is the total, cumulated number of licking frames since the beginning of the contact. A starting value for parameter α was computed using non-linear least squares estimation of the difference between our predicted load and the receiver load measured in the empirical dataset, after taking into account the constant pheromone decay with parameter k. We then used a step-by-step approach simulating the receiver load for various combinations of parameters values for $\gamma_{antennation}$, γ_{lick} and α, and selected the final parameter values that produced the lowest sum-of-square differences between predicted and empirical values. The final chosen parameter values were the following: $\gamma_{antennation} = 7.059 \times 10^{-5}$ frame^{-1}, $\gamma_{lick} = 0.021$, and α=1.057. Figure S5 shows the final fit between the predicted and empirical values.

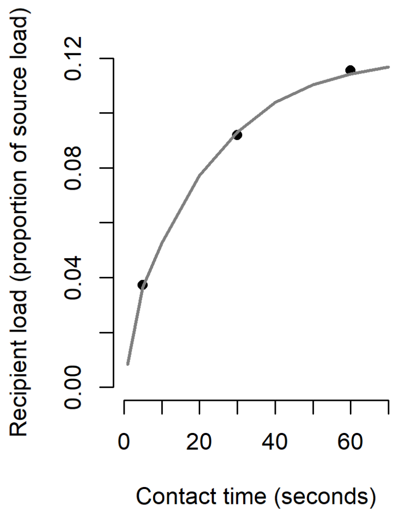

Figure S5: **Model parameterisation: diminishing transmission with longer contacts.** Black points: empirical data from Nauman (1991) [16], showing the mean receiver load after three prolonged contact durations, expressed as a proportion of the initial donor load of 250ng. Grey line: simulation outcome with parameter values $\gamma_{antennation} = 7.059 \times 10^{-5}$ frame^{-1}, $\gamma_{lick} = 0.021$ frame^{-1}, and α=1.057.

Queen bout durations

Figure S6 shows the queen bout duration distribution, pooled across all colonies, and across both stationary and travelling bouts. The distribution is right-skewed, and possesses a long tail.

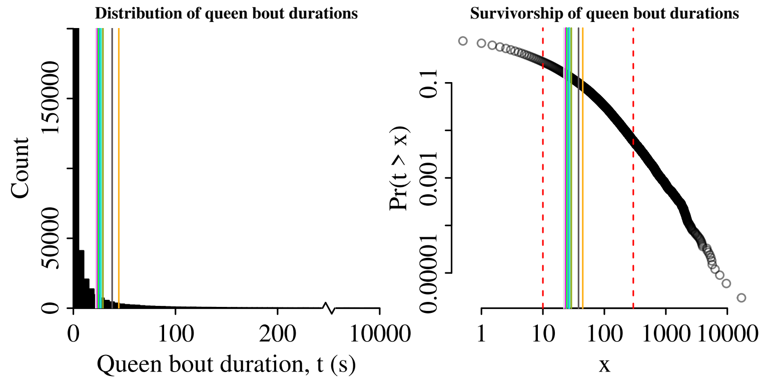

Figure S6: **Distribution of queen bouts.** (**a**) Frequency distribution of the durations of all queen bouts pooled across all colonies. Solid vertical lines indicate colony medians. (**b**) Complementary cumulative distribution (i.e., survivorship) for the pooled queen bout duration. Note, both axes have been log-transformed. Dashed red lines indicate the upper and lower cutoffs, at 10 and 305 seconds respectively.

Calculating difference vectors

The difference maps were calculated in two stages. The first was to obtain an overall vector field describing the orientations of receivers relative to the messenger. To do so, for each colony on each day, we identified all times when a worker occupied a given grid site within the messenger-centric coordinate system (x, y). We then extracted the receiver headings associated with these visits, to obtain a circular distribution, $P(\theta) \in (-\pi, \pi)$ for each (x, y) grid, and for each of the 10 colonies. This distribution was summarised by a polar vector $\vec{v} = r\bar{\theta}$, where the magnitude r was given by the mean resultant length, and the heading $\bar{\theta}$ was given by the circular mean. We then combined the ten within-colony vectors, thus producing a 'grand mean' vector (Fig. S7a-b). By sequentially averaging in this manner, we ensured equal weighting of all colonies within the overall grand mean.

The second stage was to calculate the difference in receiver orientation between pre- versus post-encounter messengers, or between post-encounter messengers with a given pheromone load and all post-encounter messengers irrespective of load. Thus, for the analysis of the effect of the queen encounter upon the messenger's subsequent encounters, we subtracted the receiver vector for pre-encounter messengers from the vector for post-encounter messengers, that is, $\Delta\vec{v}_{time} = \vec{v}_{\text{post}} - \vec{v}_{\text{pre}}$. The vector connecting the origin to the end of the reversed pre-encounter vector gives the 'difference vector', $\Delta\vec{v}_{time}$ (Fig. S7c).

In both of the analyses presented in Figure 4 in the main paper, the difference vector maps were conditioned on an additional variable; in the analysis of how a queen encounter influences the subsequent encounters of the messenger (Figure 4 a-c), this variable was a sequence of time-windows of width w =60 seconds, covering the 300 second period immediately before and immediately after the queen encounter. Difference maps were calculated for each window, by subtracting the pre-encounter vectors for the nth window before the queen encounter from nth window after it. Thus, the difference vector map for the fifth one-minute time window could be written, $\Delta\vec{v}_{241:300} = \vec{v}_{241:300} - \vec{v}_{\text{-}241:\text{-}300}$.

In the analysis of how the amount of queen pheromone a messenger carries (its load) influences encounters with other workers (Figure 4 d-f), the conditioning variable was given by the deciles of the colony load distribution. This was because differences in the number of tagged workers between colonies and across days influence the (simulated) volume of the queen pheromone that is in circulation at a given mement. To facilitate comparisons between days or between colonies, a daily load distribution was produced for each colony, and the load carried by a given messenger at a given time expressed in terms of the load deciles of this distribution. For the load distribution, we used the instantaneous loads (i.e., the frame-by-frame values) of the pre- and post-encounter messengers,

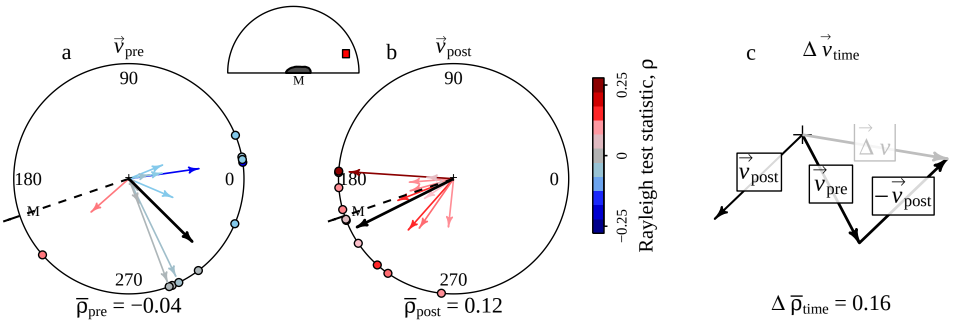

Figure S7: **Calculating difference vectors for receivers orienting towards pre- versus post-encounter messengers.** The two circular plots show the typical orientations of receivers visiting an anterior grid within the messenger-centric coordinate system, as shown by the red square in the upper panel insert. Typical receiver orientations at this site are shown for pre- and post-encounter messengers (panels **b** & **b** respectively). Each coloured arrow represents a polar vector $\vec{v} = r\bar{\theta}$ for a single colony, where the direction $\bar{\theta}$ indicates the circular mean of the receiver headings, and the magnitude r indicates the mean resultant length. Arrow colours indicate the Rayleigh test statistic, ρ, which measures the extent to which a given vector is oriented towards or away from a specified direction, as provided by the direction from the grid to the messenger (dashed black line, 'M'). The $\bar{\rho}$ values below each plot indicate the mean Rayleigh test statistic for the pre-and post-encounter messengers, where each colony contributes one value to the average. The black arrows indicate the 'grand mean' vector, obtained by vector addition of the 10 colony vectors. (c) Using vector subtraction to obtain the post-pre 'difference vector', $\Delta\vec{v}$.

obtained from the daily transmission simulations. Thus, the difference vector map for a messenger whose current load was greater than 90% of the rest of the workers in their colonies could be written; $\Delta\vec{v}_{91:100\%} = \vec{v}_{91:100\%} - \vec{v}_{1:100\%}$.
